# Diagnostic Performance of an Artificial Intelligence Model Based on Contrast-Enhanced Ultrasound in Patients with Liver Lesions: A Comparative Study with Clinicians

**DOI:** 10.3390/diagnostics13213387

**Published:** 2023-11-05

**Authors:** Marinela-Cristiana Urhuț, Larisa Daniela Săndulescu, Costin Teodor Streba, Mădălin Mămuleanu, Adriana Ciocâlteu, Sergiu Marian Cazacu, Suzana Dănoiu

**Affiliations:** 1 Department of Gastroenterology, Emergency County Hospital of Craiova, Doctoral School, University of Medicine and Pharmacy of Craiova, 200349 Craiova, Romania; cristiana.urhut@yahoo.com; 2 Department of Gastroenterology, Research Center of Gastroenterology and Hepatology, University of Medicine and Pharmacy of Craiova, 200349 Craiova, Romania; costinstreba@gmail.com (C.T.S.); adriana_ciocalteu@yahoo.com (A.C.); cazacu2sergiu@yahoo.com (S.M.C.); 3 Department of Pulmonology, University of Medicine and Pharmacy of Craiova, 200349 Craiova, Romania; 4 Oncometrics S.R.L., 200677 Craiova, Romania; madalin.mamuleanu@edu.ucv.ro; 5 Department of Automatic Control and Electronics, University of Craiova, 200585 Craiova, Romania; 6 Department of Pathophysiology, University of Medicine and Pharmacy of Craiova, 200349 Craiova, Romania; suzanadanoiu@yahoo.com

**Keywords:** artificial intelligence, contrast-enhanced ultrasound, liver tumors

## Abstract

Contrast-enhanced ultrasound (CEUS) is widely used in the characterization of liver tumors; however, the evaluation of perfusion patterns using CEUS has a subjective character. This study aims to evaluate the accuracy of an automated method based on CEUS for classifying liver lesions and to compare its performance with that of two experienced clinicians. The system used for automatic classification is based on artificial intelligence (AI) algorithms. For an interpretation close to the clinical setting, both clinicians knew which patients were at high risk for hepatocellular carcinoma (HCC), but only one was aware of all the clinical data. In total, 49 patients with 59 liver tumors were included. For the benign and malignant classification, the AI model outperformed both clinicians in terms of specificity (100% vs. 93.33%); still, the sensitivity was lower (74% vs. 93.18% vs. 90.91%). In the second stage of multiclass diagnosis, the automatic model achieved a diagnostic accuracy of 69.93% for HCC and 89.15% for liver metastases. Readers demonstrated greater diagnostic accuracy for HCC (83.05% and 79.66%) and liver metastases (94.92% and 96.61%) compared to the AI system; however, both were experienced sonographers. The AI model could potentially assist and guide less-experienced clinicians to discriminate malignant from benign liver tumors with high accuracy and specificity.

## 1. Introduction

In a clinic with a hepatobiliary profile, focal liver lesions (FLLs) are a frequent reason for evaluation [1]. Benign liver tumors are often incidental findings and have favorable evolution. The most frequently detected benign liver tumors are hepatic hemangioma, focal nodular hyperplasia and hepatocellular adenoma [2].

Primary liver cancer is the sixth most frequently diagnosed malignancy and the third leading cause of cancer death globally. In 2020, liver cancer was responsible for 906,000 new cases and 830,000 deaths [3]. 

A percentage of 5.4% of patients with extrahepatic malignancies have liver metastases at the time of diagnosis, which significantly decreases their survival [4]. Therefore, a rapid and early diagnosis of the nature of a liver tumor is required [5].

B-mode ultrasound is usually the first imaging investigation used to detect liver lesions and completes the information gathered through anamnesis and physical examination. However, the performance of greyscale ultrasound for discrimination between benign and malignant lesions is limited [6,7,8]. Contrast-enhanced ultrasound (CEUS) has better diagnostic accuracy compared to standard ultrasound (US) and performs similarly to computed tomography (CT) and magnetic resonance imaging (MRI) to rule in or rule out malignancy and also to establish the tumor type [9]. 

The Guidelines and Good Clinical Practice Recommendations for CEUS in the Liver, updated in 2020, suggest the use of CEUS as a first-line method to evaluate focal liver lesions discovered incidentally by standard ultrasound, in the absence of liver cirrhosis and in oncology patients or patients with suspected malignancy [10]. If the CEUS aspect indicates a benign tumor, further exploration is no longer necessary [10], which avoids radiation exposure and decreases the psychological burden of patients undergoing unnecessary additional investigations [10,11,12].

Nevertheless, it is worth noting that the interpretation of CEUS enhancement patterns by the examiner is subjective [13,14]. Quantitative CEUS assessment using time–intensity curves (TICs) may overcome this limitation [15]. 

In hepatology, ultrasound-based artificial intelligence has been applied to assess diffuse liver diseases and focal liver lesions [16]. Several AI methods based on CEUS have been proposed to differentiate between benign and malignant liver lesions [17,18,19,20,21]. Guo et al. [17] developed a computer-aided diagnosis (CAD) system based on three representative images from the arterial phase, portal venous phase and late phase of CEUS to classify liver lesions as benign or malignant. Deep canonical correlation analysis (DCCA) was applied on three pairs of features extracted from CEUS images, and the resulting features were provided to a multiple kernel learning (MKL) classifier. The study group comprised 93 liver tumors, of which 47 were malignant and 46 were benign. The CAD system reached an accuracy of 90.41%, a sensitivity of 93.56% and a specificity of 86.89% [17]. Turco et al. [18] developed a machine-learning (ML) approach to determine the benign and malignant nature of liver lesions in patients at risk for HCC. For this purpose, authors used short CEUS videos of 60 s that consisted of the arterial phase and partially included the portal venous phase. Both spatiotemporal features and texture features were employed. The dataset comprised 87 focal liver lesions, of which 74 were malignant (mainly HCCs) and 13 were benign. The lack of motion compensation was an undoubted advantage of the study. Another benefit of this study was that only minimal human intervention in tumor localization was required. The balanced accuracy of the ML approach to differentiate between benign and malignant tumors was 84% [18]. Wu et al. [19] used sparse non-negative matrix factorizations to automatically extract TICs from CEUS videos. Furthermore, a deep-learning classification model based on TICs was developed. The method was evaluated in a sample of 26 liver tumors. The performance parameters were as follows: 86.36% accuracy, 83.33% sensitivity and 87.50% specificity [19]. Ta et al. [20] proposed two CAD systems, one based on an artificial neural network (ANN) and one based on a support vector machine (SVM) in a multicenter study performed on 105 focal liver lesions. The effectiveness of the CAD systems for differentiating benign from malignant liver lesions was compared with that of an inexperienced and experienced observer, both blinded to the final diagnosis. The accuracy of the SVM and ANN was 81.1% and 80%, respectively. The CAD systems performed better than the inexperienced reader and similar to the experienced reader. The accuracy of both observers increased when their diagnosis was concordant with the AI assessment. The homogeneity of the lesions in the B-mode images and the TIC washout time features had the most impact in differentiating FLLs using CAD systems [20]. Also, in [21], the authors compared radiologists’ diagnostic performance with an AI method trained on a sample of 363 liver tumors and further tested on 211 cases. The AI performed as well as two experienced radiologists and showed better results than the less-experienced radiologists, represented by two residents. Assisted by AI, the diagnostic efficiency of residents increased to a level similar to that of the senior radiologists [21].

AI also showed promising results in identifying different classes of liver tumors [22,23,24,25,26]. Streba et al. [22] proposed an artificial neural network to classify five types of liver tumors: hepatic hemangiomas, fatty focal changes, HCCs, hypervascular liver metastases and hypovascular liver metastases. The development of the artificial neural network was based on CEUS TIC analysis achieved in 112 patients. The ANN registered a training accuracy of 94.45% and a testing accuracy of 87.12%, similar to results achieved by the physician [22]. Caleanu et al. [23] created a CAD system based on deep neural networks (DNN) to discriminate between five classes of liver tumors from CEUS images, with an accuracy of 88%. Liver lesions were represented by focal nodular hyperplasia (FNH), hepatic hemangiomas, HCCs, hypervascular liver metastases and hypovascular liver metastases [23]. 

Although the results of the previous literature studies discussed from the clinician’s perspective showed that AI could potentially improve the evaluation of liver tumors, there are still barriers to successfully implementing AI in clinical practice. More studies are needed to improve AI methods for assessing liver lesions. In this regard, our first goal in this study is to evaluate the importance of an AI system based on CEUS in classifying liver lesions. The second objective is to compare the performance of the AI model with the subjective analysis of two physicians in order to perform in depth tests of the AI system proposed in our previous works [27,28]. The present study is a continuation of our previous research, in which the development stages and the architecture of the proposed automatic method have been largely described [27,28]. 

The major contributions of this study are as follows: (1) inspired by the clinical practice, we integrated important clinical parameters (age, gender and the presence of an underlying liver condition) along with parameters extracted from the time–intensity curves of CEUS into an AI algorithm, providing a strategy closer to the real evaluation of liver lesions; (2) a two-stage classification of focal liver tumors was performed, as the AI system was tested for its capacity to distinguish between benign and malignant liver tumors and also for the ability to predict the diagnosis of two significant liver malignancies: hepatocellular carcinoma and liver metastases.

## 2. Materials and Methods

The present study included patients with focal liver lesions evaluated in a tertiary gastroenterology and hepatology department between January 2018 and December 2020. Based on the inclusion and exclusion criteria below, we prospectively selected 49 patients with 59 focal liver lesions. One patient was excluded due to the unsatisfactory quality of CEUS video clips. The research was approved by the Ethical Committee of the University of Medicine and Pharmacy of Craiova (36/22 April 2016). The dataset was also used to build the AI system described in [27,28] and a technical description of the investigation was presented in these works.

The criteria for inclusion were as follows: the presence of at least one focal liver lesion, CEUS examinations stored as high-quality video clips from all three vascular phases, availability of clinical information of the patient in the hospital database and final diagnosis of liver tumors established through contrast-enhanced imaging techniques or biopsy and histopathologic assessment, depending on the case. 

We excluded cases with poor-quality CEUS recordings, incomplete CEUS imaging data, indeterminate final diagnosis and previously treated liver tumors.

Simple liver cysts were included only if detected on CEUS examinations performed for other indications.

**Figure 1:**
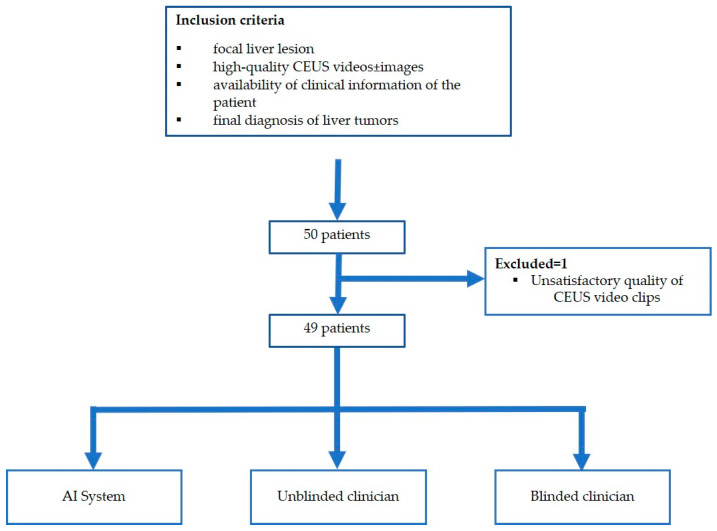
Patient enrolment and study flowchart.

The flowchart of the subject enrolment is presented in Figure 1. 

### 2.1. Standard Ultrasound and Contrast-Enhanced Ultrasound Examinations

The equipment used for standard ultrasound and CEUS investigations was a Hitachi Arietta V70 (Hitachi Ltd., Tokyo, Japan), provided with the convex probe C251. A second-generation contrast agent, SonoVue (Bracco Imaging S.p.A, Milan, Italy) was used to perform CEUS. Depending on the case, the contrast agent was administrated at a dose of 1.6/2.4 mL in an antecubital vein, followed by a 5 mL sodium chloride 0.9% flush. CEUS examinations were performed according to EFSUMB guidelines [10] by an experienced sonographer (EFSUMB level III) and stored as video clips and images from the arterial phase (10–20 s to 30–45 s), portal venous phase (30–45 s to 120 s) and late phase (120 s to 4–6 min). 

### 2.2. CEUS-Based Artificial Intelligence System for Classification of Liver Tumors

The system for the automatic classification of liver tumors is based on artificial intelligence algorithms and has three main components. The first was a segmentation component based on a U-Net segmentation deep learning model trained on our dataset as described in [27]. A second component cropped each frame according to the output of the segmentation module. This component was also responsible for extracting the TIC and the TIC parameters. Finally, the third module was a feed-forward classifier which used as an input the output of the second component together with the clinical data of the patients, represented by age, gender, presence of chronic hepatitis or liver cirrhosis. The entire system was presented in [28].

### 2.3. Image Analysis

The anonymized CEUS videos were re-evaluated independently by two hepatologists with high expertise in ultrasound and CEUS of the liver, blinded to the final diagnosis. Only one reader (unblinded reader) was aware of the clinical data. However, for a correct interpretation of CEUS findings, close to the clinical setting, both investigators knew which patients were at risk for hepatocellular carcinoma. All lesions have been interpreted according to The Guidelines and Good Clinical Practice Recommendations for CEUS in the Liver (update 2020 edition) [10]. 

Liver lesions were analyzed in terms of enhancement degree in comparison with the adjacent liver parenchyma, enhancement pattern, degree and onset of washout when present. 

The first task for readers and the AI system was to classify liver lesions as benign or malignant.

In a further stage, a specific diagnosis was predicted when possible. As HCC and liver metastases were the predominant lesions in our study, we focused mainly on their diagnosis. CEUS investigation was considered conclusive if the tumor enhancement pattern in arterial, portal venous and late phase was typical, according to the current guidelines [10]. Otherwise, if liver lesions demonstrated atypical features in CEUS or when any conclusion couldn’t be reached, the FLLs were labelled as indeterminate. 

### 2.4. Reference Standard Method

Ultrasound alone (B-mode and contrast-enhanced ultrasound) was used for liver cysts and the diagnosis of three typical hepatic hemangiomas to minimize exposure to ionizing radiation from additional examinations that were not mandatory in these circumstances. For the other benign tumors, CT or MRI was used to determine the final diagnosis.

Liver abscess was suspected on clinical and imaging methods, but the final diagnosis was confirmed intraoperatively. 

In 72.72% of cases of malignant tumors (*n* = 32), CT/MRI was the gold standard imaging modality, while for the other cases, pathological confirmation was achieved. 

### 2.5. Statistical Analysis

Clinical factors; standard ultrasound characteristics of liver tumors; enhancement patterns during the arterial, portal venous and late phases of CEUS; presence of washout; onset and intensity of washout; the final diagnosis and the method used to confirm the diagnosis were synthesized in a Microsoft Excel 2019 (Microsoft Office Professional Plus 2019, Microsoft Corporation, Washington, WA, USA) spreadsheet. Data were expressed as mean ± SD (standard deviation), percentages and frequencies. The IBM program Statistical Analysis Software Package (SPSS) for Windows version 29.0 (IBM Corporation, Armonk, NY, USA) was used for data analysis. MedCalc’s diagnostic test evaluation calculator [29] was used to determine sensitivity, specificity, accuracy, positive predictive value, negative predictive value and 95% confidence intervals for the diagnostic performance of CEUS in both readers. Interobserver agreement on the diagnosis of liver tumors between clinicians and clinicians vs. the AI system was assessed using Cohen’s kappa coefficient (κ).

## 3. Results

Forty-nine patients with fifty-nine liver lesions were enrolled in our study. The majority of FLLs were malignant (*n* = 44), with HCC being the most common (*n* = 24), followed by liver metastases (*n* = 15), cholangiocarcinoma (*n* = 4) and malignant liver adenoma (*n* = 1). Furthermore, a total of 15 benign lesions were included. The distribution of benign liver lesions was as follows: seven hepatic hemangiomas, five liver cysts, one focal nodular hyperplasia, one hepatic adenoma and one liver abscess.

Regarding gender, 31 patients were male, and 18 were women, with an age range of 38–85 years. The mean age was 67.7 ± 8.84 for men and 61 ± 10.90 for women. Risk factors for HCC, including liver cirrhosis and chronic hepatitis B or C, were identified in 51.02% of patients. In 14.3% of cases, liver lesions were developed in a background of liver steatosis (Table 1).

One target lesion was evaluated in the vast majority of the patients (85.7%). The highest percentage of lesions were above 20 mm (86.5%). Considering the distribution of the lesions according to localization, 49.2% were situated in the right hepatic lobe, 47.5% in the left hepatic lobe and 3.4% included both hepatic lobes.

Among the 59 liver lesions, 32.2% (*n* = 19) were hyperechoic, 23.7% (*n* = 14) were hypoechoic, 25.4% (*n* = 15) isoechoic, 5.1% (*n* = 3) transonic and 13.6% (*n* = 8) of them had mixed echogenicity. Most liver tumors were well-defined and had an inhomogeneous appearance (72.9%). Ancillary features favoring HCC as mosaic architecture or nodule-in-nodule appearance were present in a small percentage of cases (8.5%). The halo sign was seen in 30.05% of cases. Ultrasound features are detailed in Table 2.

### 3.1. CEUS Enhancement Patterns

#### 3.1.1. Benign Tumors

Most liver hemangiomas (*n* = 6) showed a typical enhancement pattern on CEUS: peripheral nodular enhancement in the arterial phase followed by centripetal fill-in, with no washout. Incomplete fill-in was observed in three large tumors. One case of small-sized hemangioma showed rapid and homogenous enhancement in the arterial phase with sustained enhancement into the late phase. Focal nodular hyperplasia (FNH) showed a “spoke-wheel” arterial enhancement pattern, followed by isoenhancement in the portal venous and late phase.

Regarding liver cysts, there was no contrast agent enhancement in any CEUS phases. 

**Figure 2:**
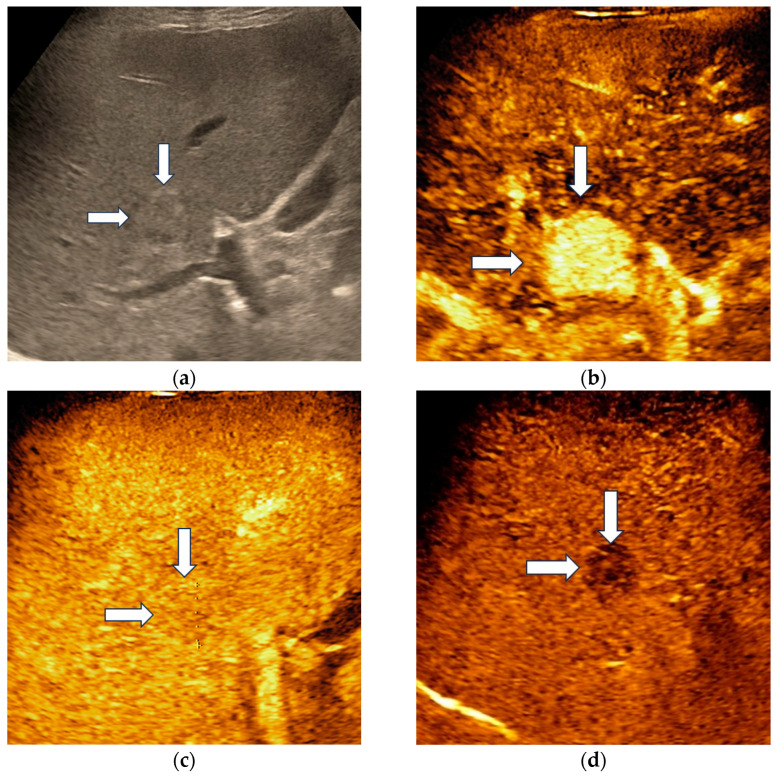
Misclassified liver adenoma by both readers and the AI system. B-mode ultrasound showed an isoechoic lesion (arrows) with a size of 23 mm, located in the right hepatic lobe (**a**). On CEUS, the lesion showed homogenous arterial hyperenhancement (arrows) (**b**), followed by isoenhancement in the portal venous phase (arrows) (**c**), with mild washout in the late phase (arrows) (**d**). Due to the presence of washout in the late phase, liver adenoma was misdiagnosed as a malignant tumor by both readers and the AI system.

Hepatocellular adenoma demonstrated homogenous hyperenhancement in the arterial phase, followed by isoenhancement in the portal venous phase and became slightly hypoenhanced in the late phase, at more than 4 min after contrast agent injection (Figure 2).

Liver abscess demonstrated a “honeycomb” appearance with enhancement of multiple septa that delimited areas with non-enhancement. In the late phase, septa became slightly hypoenhanced.

#### 3.1.2. Malignant Tumors

Typical CEUS findings of HCC represented by arterial hyperenhancement and late, mild washout were observed in 58.33% of HCC nodules (*n* = 14). Atypical enhancement patterns seen in 41.67% of all HCCs (*n* = 10) are summarized in Table 3. 

Intrahepatic cholangiocarcinomas (iCCAs) exhibited two contrast enhancement patterns in the arterial phase: heterogenous hypoenhancement (*n* = 1) and peripheral enhancement (*n* = 3). Septa enhancement was noticed in one lesion. Early and marked washout was observed in three iCCAs; however, one lesion demonstrated late contrast washout.

Malignant liver adenoma showed arterial hyperenhancement with progressive washout in the late phase.

CEUS aspects of liver metastases in the three vascular phases are summarized in Table 4.

### 3.2. Diagnostic Performance of Clinicians and the AI System

**Figure 3:**
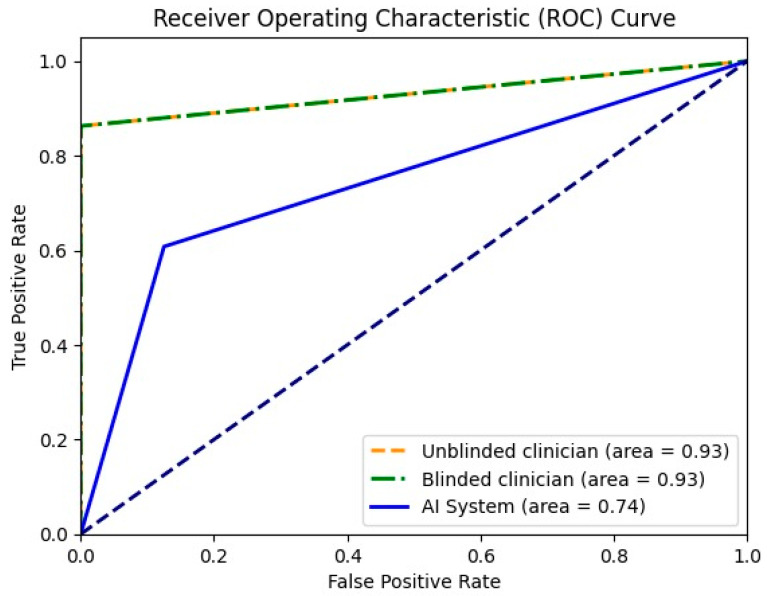
Receiver operating characteristic curve for unblinded clinician, blinded clinician and the AI system in binary mode (malignant or benign).

For the differentiation between malignant and benign liver lesions, both clinicians achieved a similar specificity of 93.33%; however, the unblinded clinician showed slightly greater sensitivity (93.18% vs. 90.91%). The AI system achieved a higher specificity compared with both readers (100% vs. 93.33%), but still had a lower sensitivity of 74% (Table 5). In Figure 3, the receiver operating characteristic curve (ROC) for all three entities is presented together with the area of the curve in numeric value. To plot the ROC, the binary classifications of all three entities (malignant or benign) were considered.

When considering diffuse, chaotic, arterial hyperenhancement followed by late, mild washout as representative for HCC, the diagnostic sensitivity was 58.33% for the unblinded clinician and 50.00% for the blinded clinician. Both clinicians achieved 100% specificity. The AI system demonstrated higher sensitivity than both clinicians for HCC diagnosis (86.91%); however, the specificity was lower (56.22%) (Table 6).

**Figure 4:**
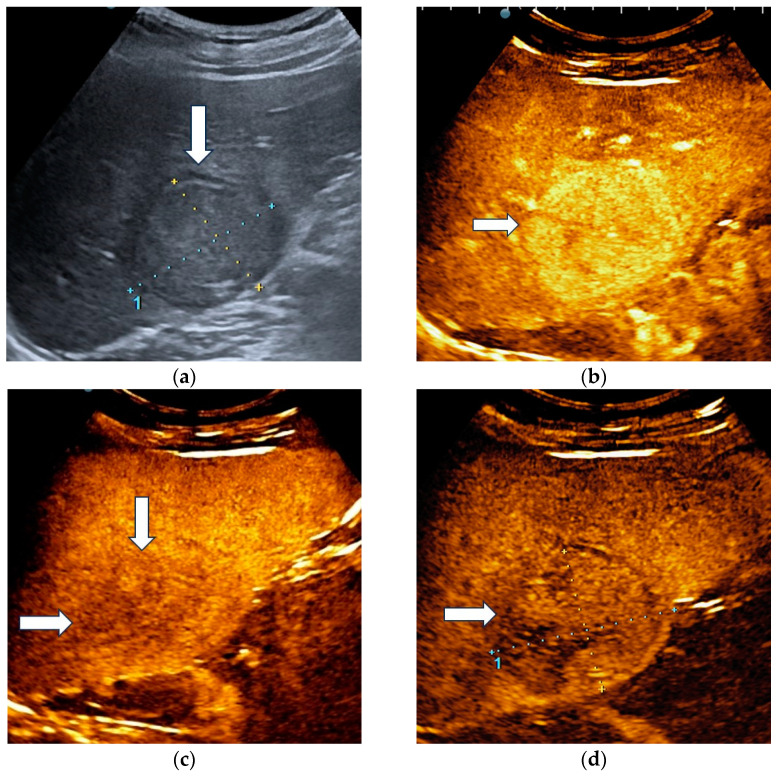
A case of hepatocellular carcinoma with typical CEUS enhancement pattern correctly classified by both clinicians and the AI system. B-mode ultrasound showed an isoechoic lesion (arrow), with a peripheral halo, sized 55/44 mm, located in the right hepatic lobe, segment VI (**a**). In the arterial phase of CEUS, the lesion showed diffuse hyperenhancement (arrow) (**b**), followed by washout in the late phase (arrows) with onset later than 2 min (**c**). At four minutes into the late phase of CEUS (arrow), the washout was still mild (**d**).

The unblinded and blinded clinicians graded six and, respectively, eight HCCs as non-HCC malignancies, from which two were developed in a non-cirrhotic liver. Three HCC nodules were categorized as indeterminate findings by the reader aware of the clinical data and were considered false negative observations. Four lesions were labelled as indeterminate by the blinded reader. The AI system correctly identified 17 HCCs (Figure 4) and misdiagnosed 6 of them as hemangiomas and one as a non-HCC malignancy. 

Regarding liver metastases, all lesions were correctly detected by the blinded reader. The unblinded clinician accurately diagnosed 14 liver metastases out of 15. He categorized as indeterminate a liver metastasis detected in a patient with chronic hepatitis B.

The AI system showed a 100% specificity; however, the sensitivity was only 22.53%, which indicates that the classifier correctly identified approximately one-fifth of the cases.

The diagnostic performance of both readers and the AI system for liver metastases is presented in Table 7.

### 3.3. Interobserver Agreement between Clinicians and AI Model

There was almost perfect agreement between clinicians (κ = 0.96) and substantial agreement between clinicians and the AI system (κ = 0.75) in differentiating malignant from benign lesions. In the multiclass liver tumors classification, there was almost perfect agreement between clinicians (κ = 0.87) and moderate agreement between the AI model and clinicians (κ = 0.42 and 0.45, respectively) (Table 8).

## 4. Discussion

Through this study, we evaluated the accuracy of an AI system based on contrast-enhanced ultrasound for identifying and classifying focal liver lesions using contrast-enhanced ultrasound examinations. 

The AI system in binary classifier mode had a sensitivity of 74% and an accuracy of 83%, indicating it is performing well on both malignant and benign lesions. NPV for benign lesions was found to be 63%. It is important to note that in the context of imbalanced data, where the number of malignant cases significantly outweighs the number of benign cases, achieving a high NPV can be particularly challenging. The AI model had a higher specificity, but a lower sensitivity when compared to clinicians; however, both physicians were experienced sonographers. 

To date, published studies in the field vary significantly in terms of the number and type of lesions and the AI algorithm used (Table 9), making it challenging to compare the results.

Gatos et al. [30] proposed an automatic algorithm for the identification and classification of 52 liver tumors using CEUS videos. The support vector machine (SVM) classification algorithm was generated based on features extracted from the time–intensity curves, demonstrating an accuracy of 90.3% in differentiating malignant from benign lesions, with a sensitivity superior to our study (93.1% vs. 74%) but a lower specificity (86.9% vs. 100%) [30].

A recently published meta-analysis [31] on the diagnostic performance of machine learning for the characterization of liver lesions (benign vs. malignant) that included 20 studies with 32.245 focal liver lesions (8 studies on standard ultrasound, 11 studies on contrast-enhanced ultrasound and 1 study on both) showed a pooled sensitivity and specificity of 81.7% and 84.8% for ML applied to ultrasound and similar results for ML based on CEUS (pooled sensitivity and specificity of 87.1% and 87%, respectively). These results were unexpected findings, as CEUS has been shown to be superior to standard ultrasound. The authors concluded that the similarities in results could be explained by the predominance of deep learning algorithms in the studies based on standard US that can provide a higher diagnostic performance [31]. 

In our study, in multiclass mode, the classifier showed a high sensitivity (86.91%) for HCC and maintained a reasonable balance between precision (PPV) and sensitivity. In the context of an imbalanced dataset, where the metastases category included a low number of lesions, achieving a sensitivity of 22.53% indicates that the classifier correctly identified approximately one-fifth of the actual cases. This result was expected given the challenge of detecting rare cases in an imbalanced dataset.

For 11 cases of HCC, all three entities agreed on the final diagnosis. For one patient with HCC, the AI system classified it correctly, while both clinicians indicated it as metastasis. Also, for two other patients with HCC, both clinicians suggested an indeterminate malignant tumor, while the AI system classified the tumor as HCC. While both clinicians tried to classify the lesion as accurately as possible, the AI system is biased towards HCC classification due to the class imbalance of the dataset. 

Shiraishi et al. [25] proposed a CAD algorithm based on microflow imaging of CEUS in 103 liver lesions and employed six artificial neuronal networks. For the classification of liver metastases, the accuracy was similar to our study (88.5% vs. 89.15%). Regarding hepatocellular carcinoma, their study showed better results in terms of accuracy (86.9% vs. 69.93%). Considering five classes of liver tumors (hemangioma, liver metastases, well-differentiated hepatocellular carcinoma, moderately differentiated hepatocellular carcinoma and poorly differentiated hepatocellular carcinoma), the accuracy decreased to 75.7% [25]. 

Clinical and laboratory data are essential in evaluating patients with an incidental focal liver lesion, as subsequent management depends on the patient’s risk factors such as a history of malignancy, liver cirrhosis or other risk factors for primary liver cancer [32]. 

In light of this, we also used clinical data such as age, gender and the presence of chronic hepatitis or liver cirrhosis in addition to the features extracted from the time–intensity curve to train the AI system. Only a few studies reported using clinical data in computer-assisted diagnosis algorithms [33,34]. Sato et al. [33] developed a deep learning model using a convolutional neural network to classify liver tumors on greyscale ultrasound. Furthermore, he gradually incorporated the following clinical information step-by-step and generated another four deep learning models: age and gender, aspartate aminotransferase and alanine aminotransferase, platelet count and albumin. The highest diagnostic performance with a sensitivity, specificity and accuracy of 100%, 92.45% and 96.3%, respectively, was achieved by the model that integrated B-mode images and all the previously mentioned data [33]. Liu et al. [34] proposed four deep-learning radiomics models for recognizing the nature of liver tumors. The models were trained with features extracted from CEUS examinations, clinical data such as the presence of underlying liver disease (HBV infection, chronic hepatitis C, liver steatosis and liver cirrhosis) and laboratory data (alpha-fetoprotein level). They included 303 patients with histopathological confirmation of liver masses diagnosis. The deep learning model trained with CEUS cines, AFP and liver disease showed the highest performance. Compared to our results, their model exhibited a higher sensitivity but lower specificity. For lesions larger than 20 mm, the AI method outperformed radiologists. In the group of lesions smaller than 20 mm, the diagnostic capability was inferior to both radiologists in the internal validation cohort; however, in the external validation cohort, it overcame only the less experienced radiologist [34].

Only a limited number of studies evaluated AI in CEUS of liver tumors using the contrast agent Sonazoid (perflubutane microbubbles) [24,26,35]. Kondo et al. [26] employed SVM classifiers to differentiate FLLs in CEUS with Sonazoid images in 98 patients. In the first stage, tumors were classified as benign or malignant with a sensitivity of 94%, specificity of 87.1% and accuracy of 91.8%. Furthermore, malignant tumors were classified as HCCs or liver metastases. For the three classes, the accuracy decreased at 84.4% for benign tumors, 87.7% for HCC and 85.7% for liver metastases [26].

In high-risk patients, the typical pattern of HCC is represented by diffuse arterial hyperenhancement and late, mild washout [10]. In our study, typical features allowed the diagnosis of HCC with 58.33% sensitivity and 100% specificity by the unblinded reader. Similar sensitivities were reported in previous studies involving larger cohorts [36,37].

However, a significant number of hepatocellular carcinomas have atypical imaging features, not meeting the criteria for the definitive diagnosis [38]. In these circumstances, histological proof is required [39]. Computer-aided diagnosis could provide a significant contribution in the differential diagnosis of hepatocellular carcinoma with unspecific features from other liver tumors. Li et al. [40] also developed a machine learning-based model using features extracted from B-mode US, arterial and portal venous phase of CEUS to differentiate atypical HCC from focal nodular hyperplasia. The automatic model achieved a lower sensitivity (76.6% vs. 94.4%) but a higher specificity (80.5% vs. 69.8%) compared with the interpretation of radiologists. The performance significantly improved when adding the AI model to the radiologist’s evaluation [40]. Huang et al. [41] proposed a computer-aided diagnosis system based on spatio-temporal features extracted from CEUS for the differential diagnosis of atypical hepatocellular carcinoma, and FNH and achieved an accuracy of 94.4%, specificity of 93.62% and sensitivity of 94.76%.

**Table 9:** Studies applying AI methods based on CEUS for liver lesions evaluation.

Author	Contrast Agent	Sample Size	Classes of FLLs	AI Method	Performance Results
**Sugimoto et al., 2010** **[24]**	Sonazoid (GE Healthcare, Oslo, Norway)	137	Hepatic hemangiomas, HCC (well-differentiated HCC, moderately differentiated HCC, poorly differentiated HCC), liver metastases	ANN	ACC of three classifications: hepatic hemangiomas—93.3%; HCC—98.6%; liver metastases—84.8%.
**Streba et al., 2012 [22]**	-	112	Focal fatty changes, hepatic hemangiomas, HCC, hypervascular liver metastases, hypovascular liver metastases	ANN	Sen: 93.2% Spe: 89.7% Training ACC: 94.45% Testing ACC: 87.12%
**Wu et al., 2014 ** **[19]**	SonoVue (Bracco, Milan, Italy)	26	Benign vs. malignant	Deep learning	Sen: 83.33% Spe: 87.50% ACC: 86.36%
**Gatos et al., 2015 [30]**	SonoVue (Bracco Imaging, Milan, Italy)	52	Benign vs. malignant	SVMs	Sen: 93.1% Spe: 86.9% ACC: 90.3% AUC: 0.89
**Kondo et al., 2017** **[26]**	Sonazoid^®^ (GE Healthcare, Oslo, Norway)	98	Benign vs. malignant Benign, HCC, liver metastases	SVM	Benign vs. malignant classification Sen: 94% Spe: 87.1% ACC: 91.8% Three classifications (benign, HCC, liver metastases) ACC: benign—84.4%; HCC—87.7%; liver metastases—85.7%
**Guo et al., 2018** **[17]**	SonoVue (Bracco Imaging, Milan, Italy)	93	Benign vs. malignant	Deep canonical correlation analysis and multiple kernel learning	Sen: 93.56% Spe: 86.89% ACC: 90.41% AUC: 0.953
**Ta et al., 2018 [20]**	SonoVue (Bracco Imaging, Milan, Italy)	105	Benign, malignant	SVM ANN	SVM Sen: 90% Spe: 71.1% ACC: 81.1% AUC: 0.883 ANN Sen: 88% Spec: 71.1% ACC: 80.0% AUC: 0.82
**Shiraishi et al., 2008, [25]**	SonoVue (Bracco Imaging, Milan, Italy)	103	hepatic hemangiomas, liver metastases, HCC (well-differentiated HCC, moderately differentiated HCC, poorly differentiated HCC)	ANN	ACC: hemangiomas—93.8%; liver metastases—88.5%; HCC—86.9%; well-differentiated HCC—79.2%; moderately differentiated HCC—50%; poorly differentiated HCC—77.8%
**Huang et al., 2020 [41]**	SonoVue (Bracco Imag-ing, Milan, Italy)	342 Data set 1: 155 FNH, 49 HCCs; Data set 2: 102 FNH, 36 HCCs	Atypical HCC vs. FNH	SVM	Sen: 94.76 Spe: 93.62% ACC: 94.4%
**Caleanu et al., 2021 [23]**	-	91	Hemangiomas, FNH, HCC, hypervascular liver metastases, hypovascular liver metastases,	Deep neural networks	ACC: 88%
**Hu et al., 2021 [21]**	SonoVue (Bracco Imag-ing, Milan, Italy)	Training set: 363; Testing set: 211	Benign vs. malignant	Deep learning models	Testing set Sen: 92.7% Spe: 85.1% ACC: 91% AUC: 0.934
**Liu et al., 2022 [34]**	SonoVue (Bracco Imag-ing, Milan, Italy)	303 Training cohort: 203; IV cohort: 50; EV cohort: 50	Benign vs. malignant	Radiomics	IV cohort Sen: 97.3% Spe: 92.3% ACC: 96% AUC: 0.969 EV cohort: Sen: 96.6% Spe: 90.55 ACC: 94% AUC: 0.957
**Turco et al., 2022 [18]**	Lumason (Bracco Imag-ing, Milan, Italy)	87	Benign vs. malignant	LR, SVM, RF, kNN	b-ACC: 0.84 Sen: 76% Spe: 92% AUC: 0.84
**Our proposed method**	SonoVue (Bracco Imag-ing, Milan, Italy)	59	Benign vs. malignant HCC, Liver metastases	CNN ± feed-forward classifier	Benign vs. malignant Sen: 74% Spe: 100% ACC: 83% HCC Sen: 86.91% Spe: 56.22% ACC: 69.93% Liver metastases Sen: 22.53% Spe: 100% ACC: 89.15%

AI: artificial intelligence; ANN: artificial neural network; ACC: accuracy; AUC: area under the curve; CNN: convolutional neural network; EV: external validation; FNH: focal nodular hyperplasia; HCC: hepatocellular carcinoma; IV: internal validation; kNN: k-nearest neighbor; LR: logistic regression; RF: random forest; SVM: support vector machine; Sen: sensitivity; Spe: specificity.

The present study has some limitations. It is important to acknowledge that our study used a relatively small dataset for training the AI system and for testing the system. This limitation is a significant constraint in AI research, since larger and more diverse datasets often produce more robust and generalizable models. This study aimed to provide a preliminary assessment of our AI system’s performance in this limited setting, and showed promising results. However, we understand that the generalizability of these findings to a broader clinical population or different medical scenarios may be limited due to the constraints of the dataset. Our group was heterogeneous in terms of tumor types, with hepatocellular carcinoma and liver metastases being the most frequent. As there was an insufficient number for some categories, such as focal nodular hyperplasia, liver abscess, liver adenoma, and cholangiocarcinoma, it was not possible to evaluate the accuracy of the AI system for their diagnosis. Secondly, we used only one type of ultrasound equipment and a single contrast agent for ultrasound. Thirdly, although we also used clinical data as input for the AI system, we consider that other valuable information collected in daily clinical practice, such as tumoral markers, should be integrated to increase performance. 

The system used here needs further validation through multicentric studies.

## 5. Conclusions

In conclusion, the proposed artificial intelligence system may serve as a second opinion to clinicians in CEUS-based evaluation of liver tumors, especially for the less experienced ones or gastroenterologists in training. Integrating clinical information and CEUS data into the AI system is a major step towards clinical applicability. Further studies involving larger cohorts are necessary to validate the effectiveness of artificial intelligence in classifying different types of liver tumors. 

## Figures and Tables

**Table 1:** Underlying liver disease in the study group.

Underlying Liver Disease	*n* (%)
➢ Liver cirrhosis	17 (34.7%)
▪ HBV	3 (6.12%)
▪ HBV + HDV	3 (6.12%)
▪ HCV	4 (8.16%)
▪ Mixed etiology	4 (8.16%)
▪ Alcohol	3 (6.12%)
➢ Chronic hepatitis C	5 (10.20%)
➢ Chronic hepatitis B	3 (6.12%)
➢ Liver steatosis	7 (14.3%)

HBV: hepatitis B virus; HCV: hepatitis C virus; HDV: hepatitis D virus.

**Table 2:** Ultrasound features of focal liver lesions.

Ultrasound Features	*n* (%)
**Number of lesions studied in each patient**	
➢ Single	42 (85.7%)
➢ 2	5 (10.2%)
➢ >2	2 (4.1%)
**Size**	
➢ Mean (mm)	51.92 ± 32.66
➢ <20 mm	8 (13.6%)
➢ 20–50 mm	26 (44.1%)
➢ >50 mm	25 (42.4%)
**Localization of the lesions**	
➢ Left hepatic lobe	28 (47.5%)
➢ Right hepatic lobe	29 (49.2%)
➢ Both hepatic lobes	2 (3.4%)
**Echogenicity of FLLs**	
➢ Hyperechoic	19 (32.2%)
➢ Hypoechoic	14 (23.7%)
➢ Isoechoic	15 (25.4%)
➢ Transonic	3 (5.1%)
➢ Mixed echogenicity	8 (13.6%)
**Echotexture**	
➢ Inhomogeneous	43 (72.9%)
➢ Homogenous	16 (27.1%)
**Delineation**	
➢ Well-defined	43 (72.9%)
➢ Ill-defined	16 (27.1%)
**Halo sign**	18 (30.5%)
**Mosaic pattern**	5 (8.5%)
**“Nodule-in-nodule appearance”**	5 (8.5%)

**Table 3:** CEUS findings of atypical HCC.

Atypical HCC Enhancement Patterns		*n* (%)
Arterial phase	Isoenhancement	1 (4.16%)
	Hypoenhancement	1 (4.16%)
	Rim enhancement	1 (4.16%)
Portal venous phase/Late phase	No washout	3 (12. 5%)
	Early or marked washout	6 (25%)

**Table 4:** CEUS aspects of liver metastases.

CEUS Phases	Category of Liver Metastases	Enhancement Pattern	*n*
Arterial phase	Hypervascular metastases	Homogenous APHE	5
		Heterogenous APHE	2
	Hypovascular metastases	Rim APHE	5
		Hypoenhancement	3
Portal venous phase/Late phase	Early and marked washout		12
	Late washout		3

APHE: arterial phase hyperenhancement.

**Table 5:** Diagnostic performance of clinicians and AI system in discriminating malignant from benign lesions.

	Sensitivity 95% (CI)	Specificity (95% CI)	Accuracy (95% CI)	PPV (95% CI)	NPV (95% CI)
**Unblinded** **clinician**	93.18%	93.33%	93.32%	97.62%	82.35%
	(81.34–98.57%)	(68.05–99.83%)	(83.54–98.12%)	(86.04–99.63%)	(60.82–93.35%)
**Blinded** **clinician**	90.91%	93.33%	91.53%	97.56%	77.78%
	(78.33–97.47%)	(68.05–99.83%)	(81.32–97.19%)	(85.73–99.63%)	(57.66–90%)
**AI system** **(binary)**	74%	100%	83% (80.57–85.43%)	100%	63%
	(69–80%)	(100%)		(100%)	(56–69%)

PPV: positive predictive value; NPV: negative predictive value; AI: artificial intelligence; CI: confidence interval.

**Table 6:** Diagnostic performance of clinicians and the AI system for HCC.

	Sensitivity 95% (CI)	Specificity (95% CI)	Accuracy (95% CI)	PPV (95% CI)	NPV (95% CI)
**Unblinded** **clinician**	58.33%	100%	83.05%	100%	77.78%
	(36.64–77.89%)	(90–100%)	(71.03–91.56%)	(76.84–100%)	(68.55–84.89%)
**Blinded** **clinician**	50.00%	100%	79.66%	100%	74.47%
	(29.12–70.88%)	(90–100%)	(67.17–89.02%)	(73.54–100%)	(66.16–81.31%)
**AI system** **(multiclass)**	86.91%	56.22%	69.93%	61.52%	84.22%
	(80.85–92.57%)	(49.69–63.45%)	(66.12- 74.59%)	(56.59–66.59%)	(76.81–89.84%)

PPV: positive predictive value; NPV: negative predictive value; AI: artificial intelligence; CI: confidence interval.

**Table 7:** Diagnostic performance of clinicians and AI system for liver metastases.

	Sensitivity (95% CI)	Specificity (95% CI)	Accuracy (95% CI)	PPV (95% CI)	NPV (95% CI)
**Unblinded** **clinician**	93.33%	95.45%	94.92%	87.50%	97.67%
	(68.05–99.83%)	(84.53–99.44%)	(85.85–98.94%)	(64.22–96.47%)	(86.33–99.64%)
**Blinded** **clinician**	100%	95.45%	96.61%	88.24%	100%
	(78.20–100%)	(84.53–99.44%)	(88.29–99.59%)	(65.94–96.67%)	(91.59–100%)
**AI system** **(multiclass)**	22.53%	100%	89.15%	100%	88.80%
	(12.49–36.52%)	(100%)	(85.66–92.18%)	(100%)	(85.17–91.97%)

PPV: positive predictive value; NPV: negative predictive value; AI: artificial intelligence; CI: confidence interval.

**Table 8:** Interobserver agreement between clinicians and the AI model using Cohen’s Kappa.

	Unblinded Clinician versus AI Model	Blinded Clinician versus AI Model	Interclinician Agreement
**Differentiation of malignant from** **benign tumors**	0.75	0.75	0.96
**Multiclass liver tumors** **classification**	0.42	0.45	0.87

## Data Availability

The data used to support the findings of this study are available upon reasonable request from the corresponding author. The artificial intelligence solution presented in this study can be used in a multicentric collaboration for testing and research purposes, after contacting the corresponding author.
